# Barriers and facilitators to help-seeking for mental health problems in prison: A qualitative interview study with incarcerated males in Northern Norway.

**DOI:** 10.1192/j.eurpsy.2023.1866

**Published:** 2023-07-19

**Authors:** L. Solbakken, R. Wynn

**Affiliations:** 1 Clinical Medicine, UiT The Arctic Univeristy of Norway; 2 Divison of Substance Use and Mental Health, University Hospital of North Norway, Tromsø, Norway

## Abstract

**Introduction:**

The prevalence of mental disorders is considerably higher among incarcerated individuals than in the general population, but this burden is not matched by a proportional use of mental health services. Studies have found that incarcerated males are reluctant to seek help for mental health problems. Gaining knowledge of factors that influence incarcerated individuals to access or avoid professional help for mental health problems is important for tailoring interventions to address the mental health needs of this population. Promoting mental health service utilization among people in prison has the potential to reduce prison suicide rates and increase institutional functioning, thereby providing safer conditions for peers and staff, promoting rehabilitation, and reducing recidivism upon release.

**Objectives:**

This study explores personal, interpersonal, and systemic aspects that motivate incarcerated individuals to approach or avoid seeking help for mental health problems. While prior studies have primarily focused on barriers to help-seeking, this study also sheds light on facilitators for seeking professional help for people in prison.

**Methods:**

Individual in-depth interviews were conducted with sixteen incarcerated males from three prisons in Northern Norway. The data analysis was inspired by Grounded Theory.

**Results:**

The majority of participants shared positive personal perspectives related to professional help-seeking, whereas the barriers were predominantly perceived to be of an interpersonal and systemic nature. Aspects that encouraged help-seeking were: regarding mental health treatment as necessary for successful rehabilitation, sufficient knowledge of when and how to contact mental health services, support from peers, having a higher ranking in the prison hierarchy, health services that are out-reaching, and prior positive experiences with professional help. The barriers to professional help-seeking were: lack of information about when and how to access mental health services, challenges with the self-referral system, perceived unavailability of services, confidentiality issues, and a prison climate that favoured a tough appearance.

**Conclusions:**

The participants appeared to be more positive to seeking professional help for mental health problems than reported in previous studies with incarcerated individuals. Interpersonal and systemic aspects were emphasized by the participants as barriers to accessing mental health services; some participants even perceived these services to be unavailable to them. The results are relevant for designing interventions to promote help-seeking for mental health problems among people in prison.

**Disclosure of Interest:**

None Declared

